# Predictive factors in spinal meningiomas – a comparative analysis with intracranial meningiomas of a high-volume skull and base center

**DOI:** 10.1007/s10143-026-04151-x

**Published:** 2026-02-07

**Authors:** Christina Fodi, Hanna Gött, Peter Paßlack, Jens Schittenhelm, Jonas Tellermann, Jürgen Honegger, Marcos Tatagiba, Felix Behling, Hannes Becker

**Affiliations:** 1 Anesthesiology and Intensive Care Medicine, University Hospital Tuebingen, Eberhard-Karls-University Tuebingen, Hoppe-Seyler Street 3, Tuebingen, Germany; 2 Department of Neurosurgery, University Hospital Tuebingen, Eberhard-Karls- University, Tuebingen, Germany; 3 Center for CNS Tumors, Comprehensive Cancer Center Tuebingen-Stuttgart, University Hospital Tuebingen, Eberhard-Karls-University Tuebingen, Tübingen, Germany; 4 German Cancer Consortium (DKTK), DKFZ partner site Tuebingen, Tübingen, Germany; 5 Interdisciplinary Division of Neuro-Oncology, University Hospital Tuebingen, Eberhard-Karls-University Tuebingen, Tübingen, Germany; 6 Hertie Institute for Clinical Brain Research, Tuebingen, Germany; 7 Department of Neuropathology, Institute of Pathology and Neuropathology, University Hospital Tuebingen, Eberhard-Karls University, Tuebingen, Germany; 8 Department of Neurosurgery, University Hospital Augsburg, Augsburg, Germany; 9 Translational Oncological Neurosurgery, Faculty of Medicine, University Augsburg, Augsburg, Germany

**Keywords:** Spinal meningioma, Predictive factors, Simpson grading, Recurrence

## Abstract

Microsurgical resection remains the gold standard in the treatment of spinal meningiomas. Available data suggest more favorable clinical outcomes compared to intracranial meningiomas. This study aims to enhance risk stratification and optimize treatment strategies by analyzing clinical and histopathological predictive factors for spinal meningiomas in a large single-center cohort. We present the analysis of 217 spinal meningiomas of our institutional meningioma cohort treated between October 2003 and March 2017. Clinical parameters of the patients such as sex, age, location of the meningioma and extent of resection were recorded besides histological features. Other parameters included follow-up and recurrence. The outcome parameters for spinal meningiomas were evaluated and compared with 1767 intracranial meningiomas. Among 217 spinal meningiomas, 80.37% (*n* = 172) were completely resected. The majority (96.31%, *n* = 209) were classified as CNS WHO grade 1. In contrast, the proportion of WHO grade 1 among intracranial meningiomas was 78.21% (*n* = 1382, *p* < 0.0001). Patients with spinal meningiomas had a lower recurrence rate with 6.91% (*n* = 15), versus 23.94% (*n* = 423) of patients with intracranial meningiomas (*p* < 0.0001). Multivariate analysis identified Simpson resection grade (*p* < 0.0001) and higher WHO grade (*p* = 0.0156) as independent prognostic factors for risk factors for progression in spinal meningiomas. For intracranial meningiomas, male gender (*p* = 0.0106) and resection of a recurrent tumor (*p* < 0.0001) were additionally identified as independent negative prognostic parameters. Spinal meningiomas are associated with a more favorable prognosis compared to their intracranial counterparts. The most significant and modifiable prognostic factor is the achievement of gross total resection. Given the technical complexity of surgery, spinal meningiomas should preferably be treated in high-volume centers with experienced surgeons to achieve this goal while minimizing morbidity and ensuring durable tumor control.

**Supplementary information:**

The online version contains supplementary material available at 10.1007/s10143-026-04151-x.

## Introduction

Meningiomas are the most common primary tumors of the central nervous system and account for approximately one-third of intracranial neoplasms. Most scientific interest has focused on intracranial meningiomas, resulting in detailed insights into their clinical presentation, histopathology, and molecular landscape. Spinal meningiomas represent with 25–45% the most common intraspinal neoplasm [1, 2], most frequently in the area of the thoracic spine [3, 4]. Despite the mostly benign histology features of spinal meningiomas, patients harbor relevant clinical symptom burden including pain, allodynia, hypesthesia or motor impairment [5, 6]. Of note, only about 8% of all meningiomas are located in the spinal column [7].

According to the current guidelines, small and asymptomatic lesions can be managed by wait and scan strategy, symptomatic lesions are mainly treated by microsurgical resection preserving neurological function and integrity [8–12]. Surgical treatment is associated with numerous difficulties including adhesions to crital structures, arachnoidal infiltration [13] and ventral tumor location. However, higher-grade spinal meningiomas are rare [14] and remain poorly characterized clinically. These clinical characteristics underscore the urgent need to recognize spinal meningiomas as a distinct disease entity, rather than merely extrapolating from the intracranial environment. Recent molecular profiling has reinforced this perspective [15, 16].

In summary, underlining clinical and histopathological factors influencing outcomes of spinal meningiomas have not been conclusively investigated in a real-world setting, yet.

Spinal meningiomas merit dedicated investigation to improve patient surveillance and prognosis. Besides preoperative patient’s symptom burden in spinal meningiomas, postoperative morbidity in spinal cases is sometimes higher than in some intracranial cases underscoring the value of predictive clinical and molecular markers to guide surgical treatment decisions.

Recently new insights have been gained into molecular changes whose effects on recurrence risk, growth dynamics and response to therapy are still poorly understood. In light of these considerations, spinal meningiomas should not be regarded as a mere extension of intracranial disease. Instead, they represent a clinically significant and biologically distinct subgroup of meningiomas. Dedicated studies focusing on their molecular, clinical, and anatomical characteristics are essential to improve risk stratification and optimize management strategies. Therefore, we present the data of a comprehensive single-center cohort with a focus on clinical and pathological features influencing the risk of recurrence. Our data provides implications for improving treatment and surveillance algorithms of spinal meningiomas.

## Materials and methods

We considered the consecutive series of all patients with spinal meningiomas that were surgically treated in our institution in the period from October 2003 to March 2017. For this retrospective analysis we collected the following clinical and histopathological data: sex, age, tumor localization, WHO grade (according to the WHO classification 2016 [17]) and histological subtype, neurofibromatosis type 2, tumor status (primary or recurrent), extent of resection and MIB-1 expression as proliferation marker. Additional progression-free survival and recurrence rate were analyzed. The data were collected by reviewing electronic treatment records.

We used archived routine MIB1 stainings from the institute of neuropathology to calculate quantitative immunopositivity of representative digital images, using Image J software (Version 1.51j8, NIH, Bethesda, MD, USA) and the plugins Bio-Formats (Release 5.4.1; Open Microscopy Environment, Madison, NJ, USA) and ImmunoRatio (Version 1.0c, Institute of Biomedical Technology, University of Tampere, Finland).

We excluded cases with missing consent for the scientific use of clinical data or incomplete clinical records, so that out of a total of 2168 cases, 1984 were included in the evaluation. No imputation of missing data was done.

All statistical analyses were carried out with JMP^®^ Statistical Discovery Software, version 15.1.0 (SAS Institute Inc., Cary, NC; 1989). Univariate comparisons were assessed using Pearson’s chi-squared and log-rank tests, whereas multivariate analysis was performed with the Wald test. Kaplan–Meier analysis was done for univariate prognostic assessment using the log-rank test. Results were regarded as significant at a level of α < 0.05.

This study was performed in compliance with the ethical principles of the Declaration of Helsinki (1964) and its later amendments, and with the regulations of the relevant institutional and national research committees. Ethical approval was obtained from the local Clinical Ethics Committee of our institution, Baden-Wuerttemberg, Germany (approval no. 191/2021BO2 and amendment).

## Results

### Cohort characteristics

Out of all 1984 included meningiomas, 217 (10.94%) correspond to spinal and 1767 (89.06%) to intracranial localization. A proportion of 76.49% (*n* = 166) of spinal meningiomas occurred in female patients and 23.51% (*n* = 51) in male patients. In comparison, the group of intracranial meningiomas reveals a sex distribution of 70.57% (*n* = 1247) in females and 29.43% (*n* = 520) in males (*p* = 0.0688). The mean age of patients with a spinal meningioma was 59.98 years (8.28–90.96 years). In patients with intracranial meningiomas the mean age was 56.79 years ranging from 3.83 to 88.93 years (*p* = 0.0023).

Of the 217 cases, 201 (92.63%) were primary tumors and only 16 (7.37%) were recurrencies. Among intracranially resected meningiomas, there was a higher proportion of recurrent meningiomas at 13.5% (*n* = 238, *p* = 0.0112). There were 27 spinal meningiomas in the cohort that occurred in patients with neurofibromatosis type 2 (12.44%). The remaining 190 spinal meningiomas (87.56%) occurred sporadically. In the group of patients who received resection of intracranial meningiomas significant fewer cases (5.60%, *n* = 99) were associated with neurofibromatosis type 2 (*p* < 0.0001).

**Fig. 1:**
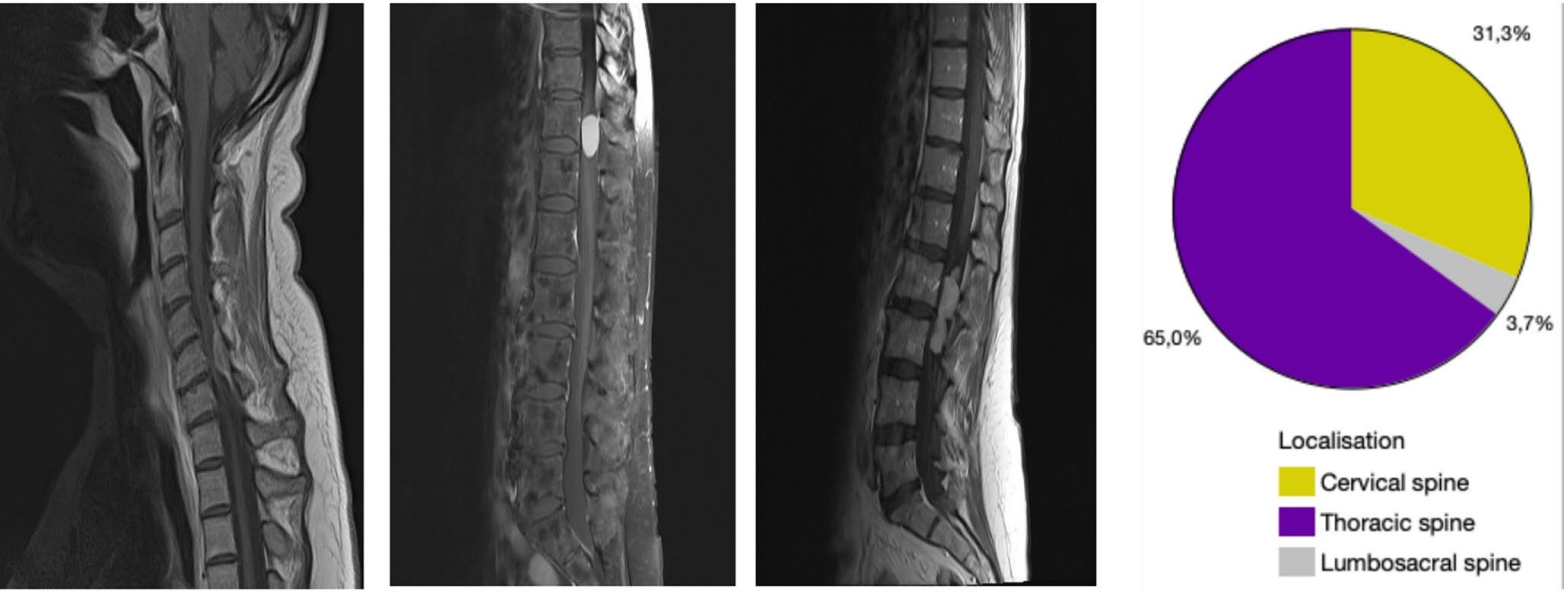
Spinal meningiomas of the cervical, thoracic and lumbar spine with graphical representation of the distribution in a pie chart

Most of spinal meningiomas were located in the thoracic spine (64.98%, *n* = 141), while 31.34% (*n* = 68) were diagnosed in the cervical and a minority of 3.69% (*n* = 8) in the lumbar spine (Fig. 1).

**Fig. 2:**
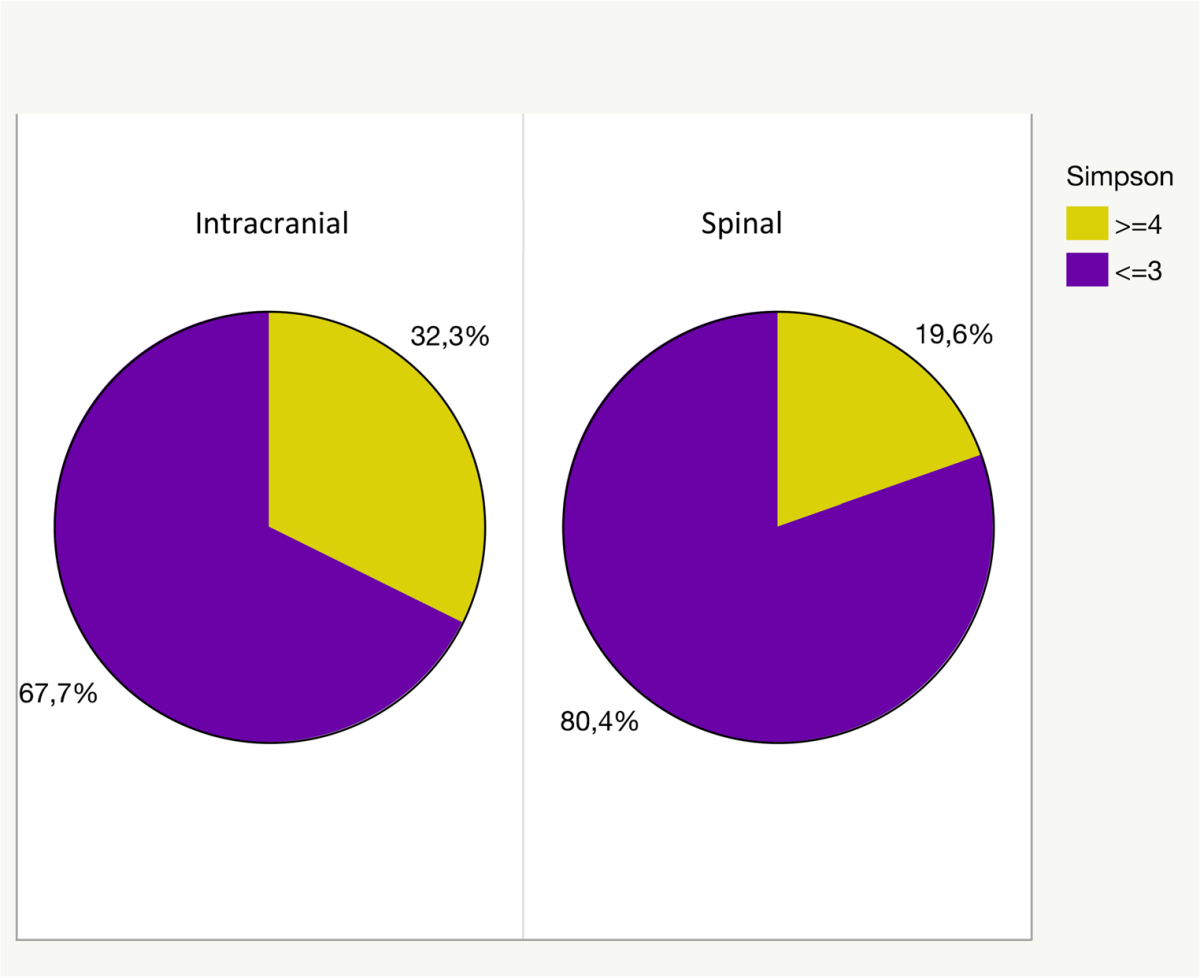
Graphical representation of the degree of resection according to Simpson of intraspinal and intracranial meningiomas

Macroscopical complete resection, defined by application of Simpson grading (grade ≤ 3) was achieved in 80.37% (*n* = 172) of spinal meningiomas. Only 19.63% (*n* = 42) were resected according to Simpson grade 4. None of the spinal meningiomas were biopsied (Simpson grade 5; Fig. 2). 67.69% of intracranial meningiomas were resected according to Simpson grade 1–3, conferring complete resection (Fig. 2). The rate of resected meningiomas with Simpson grade 4 or 5 was 32.31% (*p* = 0.0002, see Fig. 2), mainly based on complex skull and base locations.

**Fig. 3:**
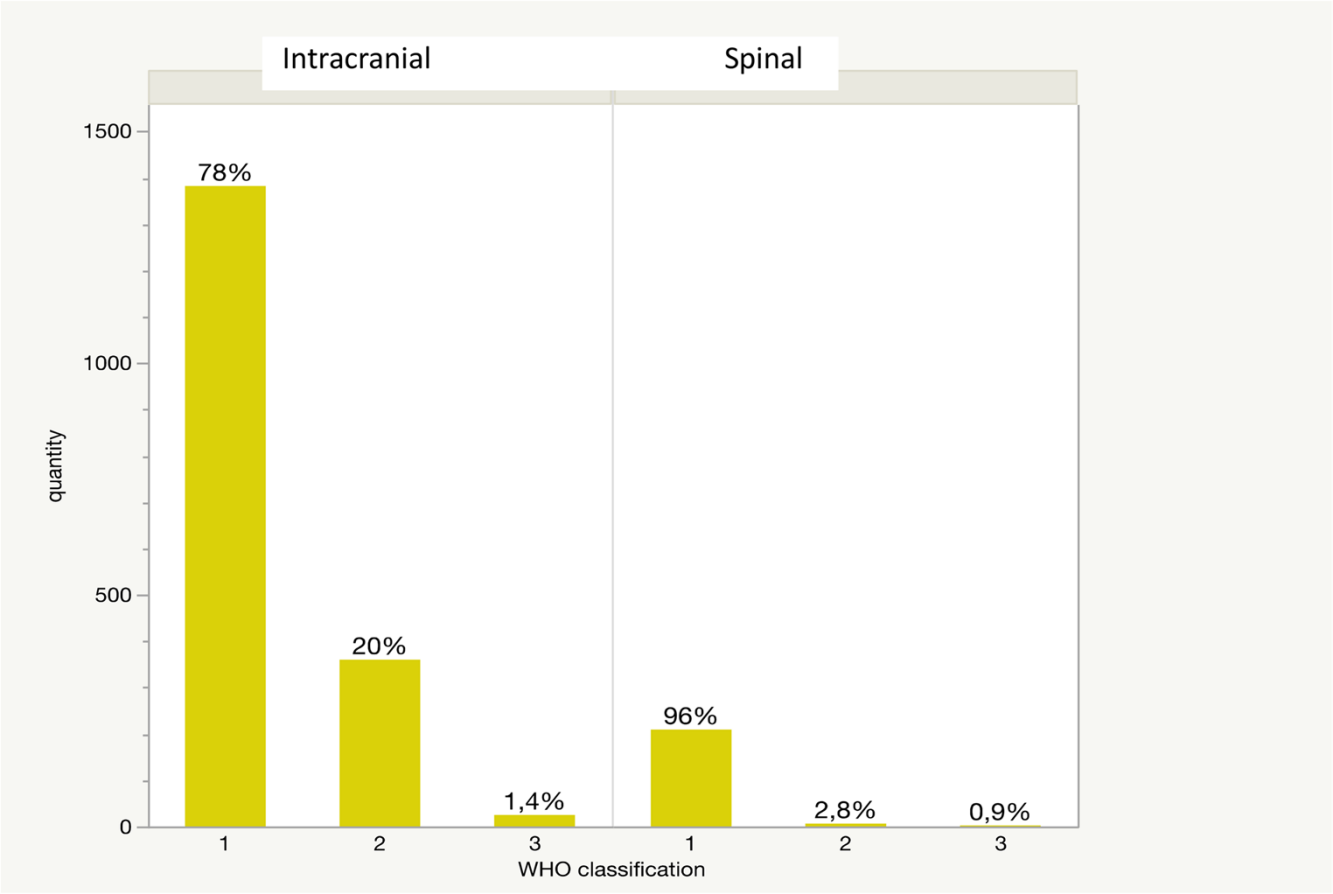
WHO grade distribution of spinal and intracranial meningiomas

Considering the assignment according to the WHO classification, 96.31% (*n* = 209) of spinal meningiomas were classified as grade 1. Only 2.77% (*n* = 6) and 0.92% (*n* = 2) corresponded to WHO grade 2 and 3 respectively, based on true atypia (Fig. 3). The histological work-up of all intracranial meningiomas revealed an allocation of 78.21% to WHO grade 1, 20.37% to WHO grade 2 and 1.42% to WHO grade 3 (see Fig. 3), demonstrating a clear statistical difference (*p* < 0.0001).

**Fig. 4:**
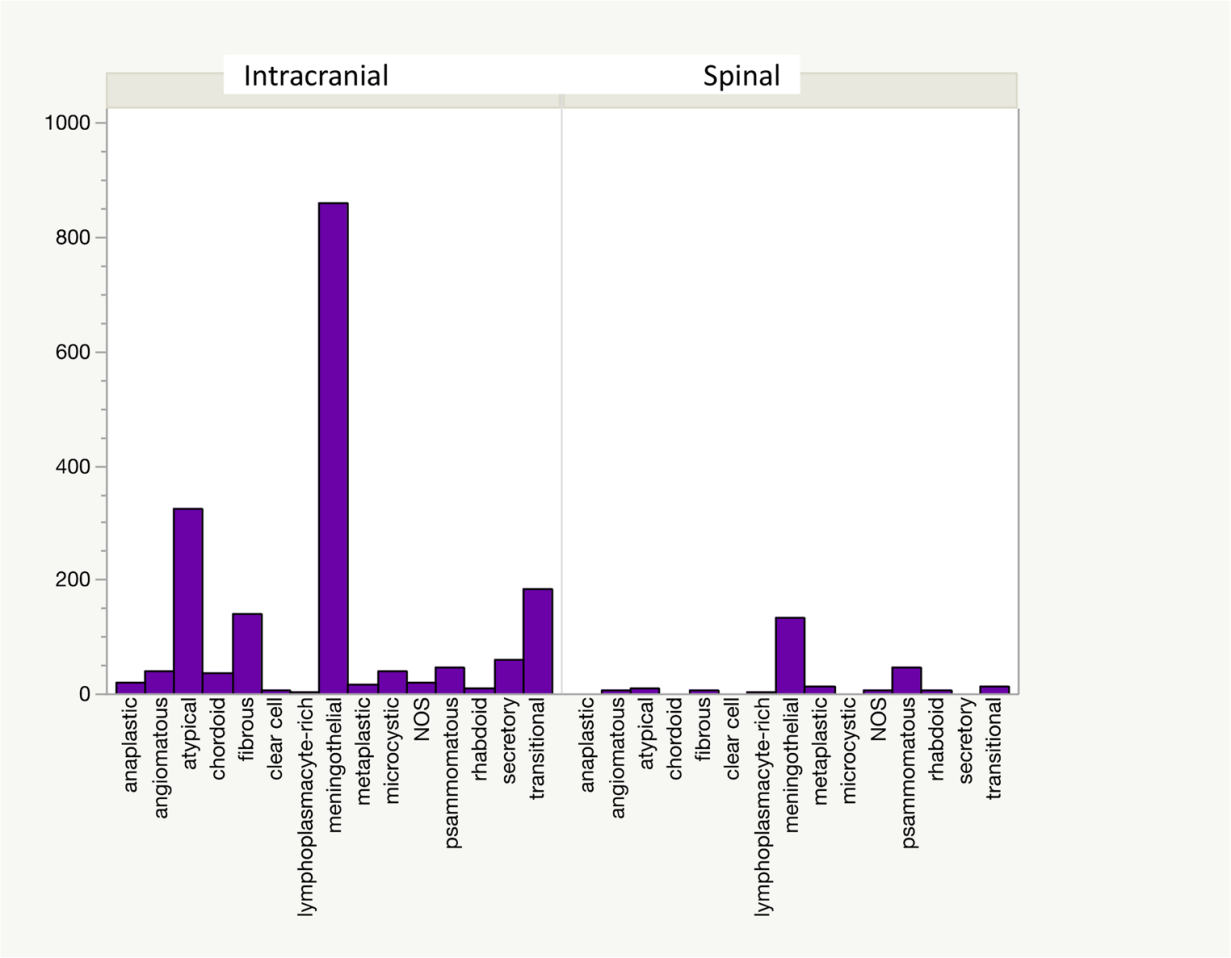
Distribution of histological subtypes of spinal and intracranial meningiomas

A further breakdown of the results by histologic subtype revealed that the meningothelial subtype was most common in both groups. A detailed presentation of all subtypes can be seen in Fig. 4.

Immunohistochemical analysis of the MIB-1 proliferation index, indicative for proliferating tumor cells, showed a mean value of 2.72% (0–10.7.7%) for spinal meningiomas. The evaluation of intracranial meningiomas yielded a slightly higher mean value of 2.98%. However, a clearly higher range of 0–34.1.1% was observed here. Comparison of the two groups did not reveal any statistically significant difference (*p* = 0.2265). An overview of all clinical data can be found in Table 1.

**Table 1:** Tabular listing of collected clinical data. The asterisk indicates statistical significance

	Spinal	Intracranial	*p*-value
Sex - Female - Male	*n* = 166 76.49% *n* = 51 23.51%	*n* = 1247 70.57% *n* = 520 29.43%	0.0688
Age (mean value and range)	59.98 years (8.28–90.96 years)	56.79 years (3.83–88.93 years)	0.0023*
Tumor status - Primary - Recurrent	*n* = 201 92.63% *n* = 16. 7.37%	*n* = 1529 86.53% *n* = 238 13.47%	0.0112*
Neurofibromatosis type 2 - Yes - No	*n* = 27 12.44% *n* = 190 87.56%	*n* = 99 5.60% *n* = 1668 94.40%	< 0.0001*
Grade of resection - Simpson grade ≤3 - Simpson grade ≥4	*n* = 172 80.37% *n* = 42 19.63%	*n* = 1169 67.69% *n* = 558 32.31%	0.0002*
CNS WHO grade - 1 - 2 - 3	*n* = 209 96.31% *n* = 6 2.77% *n* = 2 0.92%	*n* = 1382 78.21% *n* = 360 20.37% *n* = 25 1.42%	< 0.0001*
MIB-1 index (mean value and range)	2.72% (0–10.7.7%)	2.98% (0–34.1.1%)	0.2265
Follow-up (mean value and range)	35.94 months (1.25–209.82.25.82 months)	45.51 months (1.16–216.49.16.49 months)	
Recurrence - Yes - No	*n* = 15 6.91% *n* = 161 74.19%	*n* = 423 23.94% *n* = 1162 65.76%	< 0.0001*

### Outcome and follow-up

On average, the follow-up was 35.94 months, ranging from 1.25 to 209.82 months for spinal tumors compared to 45.51 months (1.16–216.49.16.49 months) for intracranial meningiomas.

The recurrence rate for spinal meningiomas was 6.91% (*n* = 15), while 23.94% (*n* = 423) of patients with intracranial meningiomas developed a recurrence. The more frequent recurrences in intracranial meningiomas proved to be statistically significant (*p* < 0.0001). However, it was striking that 18.89% (*n* = 41) of spinal meningiomas were missed during follow-up. For intracranial meningiomas, the percentage was only 10.30% (*n* = 182). The detailed data can be seen in Table 1.

**Fig. 5:**
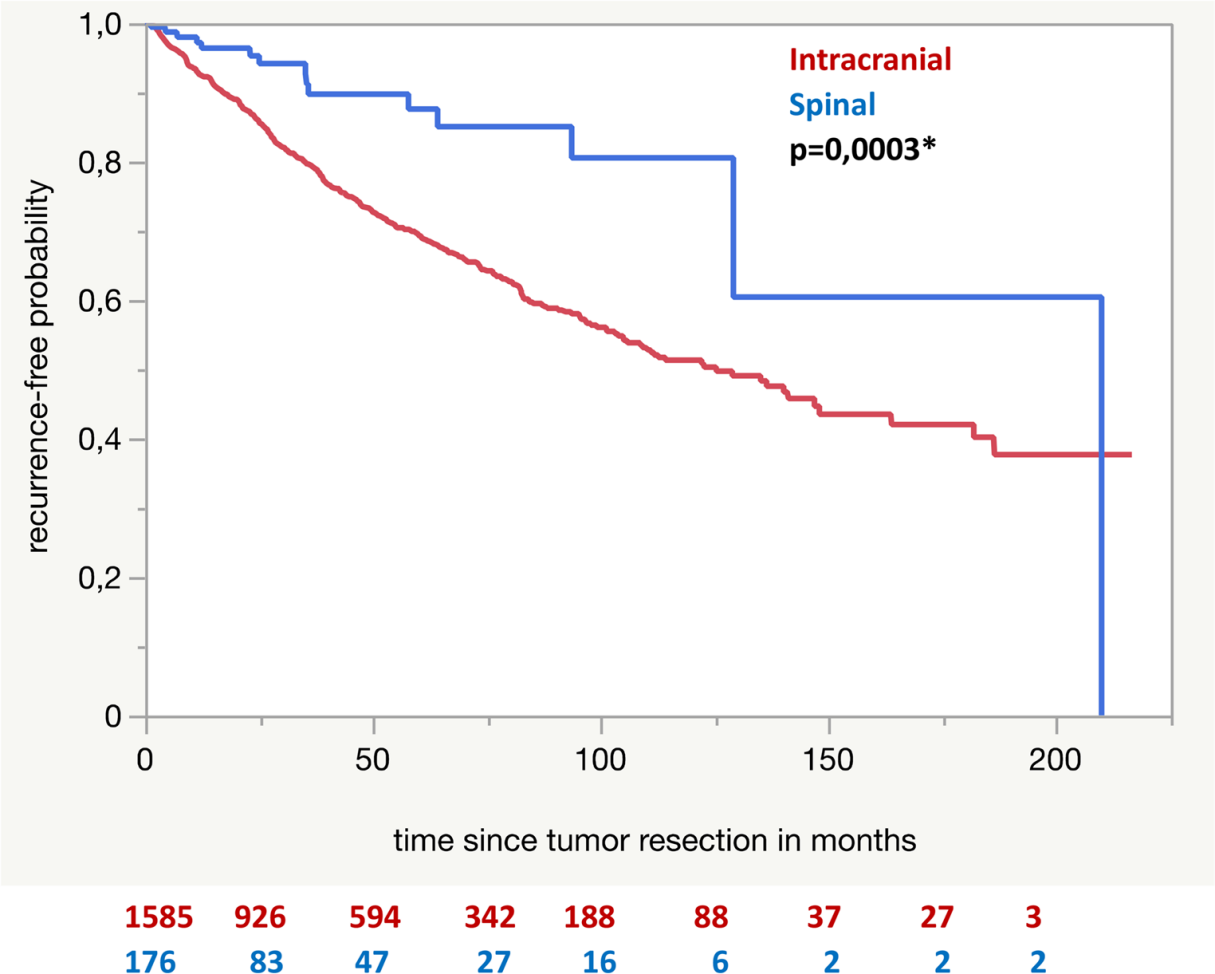
Kaplan-Meier analysis for recurrence free survival of spinal and intracranial meningiomas. The asterisk indicates statistical significance

Progression-free survival analysis demonstrated a significantly longer recurrence-free survival for spinal tumors than for intracranial tumors (*p* = 0.0003, see Fig. 5).

Multivariate analysis was performed and identified independent risk factors for tumor recurrence. Higher Simpson resection grade (*p* < 0.0001) and higher WHO grade (*p* = 0.0156) were identified as independent negative prognostic parameters for progression in spinal meningiomas. In contrast male sex (*p* = 0.0106), higher WHO grade (*p* < 0.0001) and resection of a recurrent tumor (*p* < 0.0001) were all independently associated with worse prognosis in intracranial meningiomas in addition to the negative prognostic impact of high Simpson grade (*p* < 0.0001). All parameters are visualized in Table 2.

**Table 2:** Multivariate analysis of risk factors for the development of recurrence. The asterisk indicates statistical significance

	Parameter	Risk ratios (95% CI)	*p*-value
Intracranial meningiomas	Sex - Male	1.3 (1.1–1.6)	0.0106*
	Tumor status - Recurrent	2.9 (2.3–3.6)	< 0.0001*
	WHO classification - Higher grade	1 vs. 2 2.8 (2.3–3.5) 1 vs. 3 17.7 (10.7–29.4) 2 vs. 3 6.3 (3.8–10.2)	< 0.0001*
	Simpson classification - ≥4	2.3 (1.9–2.9)	< 0.0001*
Spinal meningiomas	Sex - Male	2.2 (0.6–7.8)	0.2154
	Tumor status - Recurrent	1.2 (0.3–5.2)	0.7600
	CNS WHO grade - Higher grade	1 vs. 2 7.6 1 vs. 3 38.6 (2.4–613.4.4.4) 2 vs. 3 5.1	0.0156*
	Simpson classification - ≥4	19.0 (3.8–96.6)	< 0.0001*

Since both WHO grade and extent of resection were identified as independent negative prognostic factors in both subgroups, we additionally performed a logistic regression analysis of these two parameters for spinal and intracranial meningiomas. In patients with intracranial meningiomas, both extent of resection and WHO grade showed a strong and independent association with recurrence. Radical resection (Simpson grade ≤ 3) was associated with a significantly reduced risk of recurrence (OR 0.24, 95% CI 0.19–0.31; *p* < 0.0001), whereas WHO grade 2 and WHO grade 3 tumors demonstrated a stepwise increase in recurrence risk compared with WHO grade 1 tumors (all *p* < 0.001). In spinal meningiomas, the extent of resection remained the most relevant predictor of recurrence, with Simpson grade ≤ 3 being associated with a markedly lower likelihood of recurrence (OR 0.06, 95% CI 0.01–0.22; *p* < 0.0001). The WHO classification showed modest association, with statistical significance was mainly driven by WHO grade 3 tumors, while discrimination between WHO grades 1 and 2 did not meet statistical significance (supplementary Table 3).

Based on the high number of WHO grade 1 meningiomas in both localization groups, a logistic regression analysis of the subgroup of WHO grade 1 meningiomas was additionally performed. In intracranial WHO grade 1 meningiomas, multivariate logistic regression demonstrated that the extent of resection (Simpson ≤ 3), sex, and tumor status (primary vs. recurrent) were all independent predictors of recurrence (all *p* ≤ 0.001). Thus, recurrence risk in intracranial WHO grade 1 tumors is influenced by both surgical and patient- or tumor-related factors. In contrast, in spinal WHO grade I meningiomas, only the extent of resection remained statistical significantly associated with recurrence (*p* = 0.0006), whereas sex and primary versus recurrent tumor status showed no independent effect (supplementary Table 4).

To assess the confounding effect of adjuvant radiotherapy on recurrence, the parameter was included in multivariate logistic regression models. In intracranial meningiomas, adjuvant radiotherapy was independently associated with recurrence (*p* < 0.0001). However, inclusion of radiotherapy did not attenuate the effects of extent of resection, WHO grade, sex, or tumor status, indicating that the main results were robust and not driven by treatment-related bias. In spinal meningiomas, adjuvant radiotherapy could not be reliably evaluated due to low event numbers and did not influence the association between extent of resection and recurrence, which remained the sole independent predictor (supplementary Table 5).

## Discussion

Adequate treatment of spinal meningiomas including pragmatic surveillance strategies are of high clinical relevance. Our findings support the existing literature that microsurgical resection offers a highly effective treatment strategy with excellent long-term tumor control including low recurrence rates [3, 8, 18–23]. This is in line with our results, which demonstrated a recurrence rate of only 6.91% in spinal meningioma, in comparison to 23.94% observed in intracranial meningiomas surgically treated in our center (*p* < 0.0001). Moreover, shorter follow-up in the spinal meningioma cohort compared to intracranial meningiomas has been described by other authors, too [23]. This might be caused by the lack of postoperative deficits and good long-term results, mainly caused by increased number of radical resections and low experienced morbidity. As a result, patients might reveal decreased compliance in regard of clinical and radiological follow-ups. We observed that spinal meningioma patients with subtotal resection, corresponding to Simpson grade 4, more frequently developed tumor recurrence. However, recurrence rates were less frequent than in intracranial meningiomas who underwent incomplete resection [4, 24]. Complete resection (Simpson grade ≤ 3) was the most crucial modifiable factor associated with more favorable recurrence-free survival in spinal meningiomas, reaching statistical significance in the multivariate analysis. We confirmed this finding in additional logistic regression analyses. Here, subtotal resection was consistently shown to be an independent negative prognostic factor for recurrence in spinal meningiomas (supplementary Tables 3–5). Across all analyses, the extent of resection emerged as the most robust and consistent predictor of recurrence, underscoring the relevance of surgical radicality in particular in spinal meningioma management. Importantly, subgroup analyses restricted to WHO grade 1 meningiomas revealed fundamental location-specific differences. In intracranial WHO grade 1 tumors, recurrence was independently associated not only with the extent of resection but also with sex and tumor status (primary vs. recurrent), indicating a multifactorial recurrence profile even within histologically benign lesions. In contrast, in spinal WHO grade 1 meningiomas, the extent of resection was the sole independent predictor of recurrence, suggestive that recurrence in this subgroup is predominantly determined by surgical factors rather than further clinical and histopathological features (supplementary Table 4). This is in line with prior studies highlighting the central role of radical resection in spinal meningioma management [25–27]. However, it should be noted that in modern surgery, considering only the degree of resection according to Simpson, is no longer adequate, which is also widely discussed in the literature. Further parameters include postoperative MRIs to determine residual tumor volume as well as histological and immunohistochemical parameters (such as WHO grade, Ki-67/MIB-1 indices) [28–31].

Additionally, we demonstrate that the recurrence rate in the group of intracranial meningiomas in comparison to spinal meningiomas is significantly higher. However, it must be noted that the group of intracranial meningiomas was not stratified according to anatomical sub-localizations. A comparison of the recurrence rates described in the literature for intracranial vs. spinal meningiomas also shows a clear difference to the disadvantage of intracranially localized tumors [32–34]. Notably, our cohort included a large portion of skull base meningiomas, which notoriously are more difficult to resect radically, which may have contributed to higher recurrence rates compared to spinal tumors. Of note, logistic regression analyses revealed that adjuvant radiotherapy was strongly associated with recurrence in intracranial meningiomas but did not interfere with the observed effects of surgical radicality or tumor biology, while recurrence in spinal meningiomas remained predominantly determined by extent of resection (supplementary Table 5).

Depending on the extent of resection and the WHO grade of spinal meningiomas, follow-up intervals with imaging and clinical examination can be adjusted, potentially allowing for a less intensive surveillance schedule than commonly recommended for intracranial tumors.

This could ensure reliable monitoring due to better patient compliance, as symptom-free patients would not have the feeling of constantly undergo surveillance examinations. It would still be possible to detect a recurrence at an early stage when subsequent standardized follow-up intervals of low-risk spinal meningiomas. The current recommendations for follow-up examinations are based on expert recommendations, with no distinction between spinal and intracranial meningiomas [22]. However, this should be validated through larger prospective cohorts in a multi-center approach, to differentiate follow-up managements for spinal meningiomas.

It is possible that spinal meningiomas are detected early, often as an incidental finding, as imaging of the spine is more frequently carried out in the routine due to other common complaints. This could create better conditions for resection, as the tumors may not yet have infiltrated other structures to the same extent as intracranial meningiomas. Infiltration of the central nervous system is very rare. There are only isolated case reports of intramedullary meningiomas [35, 36]. This favorable circumstance of extremely rare infiltration of the CNS may therefore also explain the better PFS compared to intracranial meningiomas.

A higher WHO grade and subtotal resection have been shown to be negative independent factors influencing the risk of recurrence in spinal meningiomas in the multivariate analysis. This has been shown by other authors, too [23, 25, 37, 38]. Across both subgroups, the extent of resection proved to be the most robust and consistent predictor of recurrence. While WHO grade provided strong prognostic stratification in intracranial meningiomas, its impact in spinal meningiomas was largely confined to high-grade tumors. The prognostic impact of the WHO classification differed substantially between locations. In intracranial meningiomas, WHO grade showed a strong, stepwise association with recurrence, reflecting increasing biological aggressiveness from WHO grade 1 to grade 3. In contrast, WHO grading demonstrated limited discriminatory capacity in spinal meningiomas, with prognostic relevance largely confined to WHO grade 3 tumors, while WHO grades 1 and 2 did not differ in regard of recurrence. This observation is consistent with the more indolent clinical behavior and lower recurrence rates, reported for spinal meningiomas. Nevertheless, patients with spinal meningiomas benefit from the fact that the majority of tumors can be classified as WHO grade 1 in itself and may partially explain their better clinical behavior compared to intracranial meningiomas. Although molecular analyses were not included in this cohort, emerging studies suggest that genetic and epigenetic profiles may offer additional predictive and prognostic value in both spinal and intracranial meningiomas [39]. Recent molecular profiling has reinforced this perspective. Ricklefs et al. showed that spinal meningiomas can be divided into two distinct genetic and epigenetic classes, demonstrating clear heterogeneity within this group [15]. In parallel, Hua et al. identified two predominant molecular subtypes: NF2-mutated tumors, which occur more frequently in the thoracic spine and are strongly associated with female gender, and AKT1-mutated tumors, which are predominantly present in the cervical spine and are often located ventral to the spinal cord [16]. These findings highlight the biological uniqueness of spinal meningiomas but are suggestive for molecular correlations between clinical and anatomical distribution. However, current molecular classifiers do not clearly distinguish between anatomical regions. The inclusion of molecular diagnostics in future studies could enable more differentiated risk stratification and tailored follow-up regimes. However, in order to reduce tumor recurrences and preserve neurological functions, it remains important to achieve radical resection. It is the only prognostic factor that can be directly influenced so far. This study provides a direct, parallel comparison of intracranial and spinal meningiomas using location-specific uni- and multivariable analyses. By restricting subgroup analyses to WHO grade 1 tumors, we demonstrate for the first time that recurrence risk within histologically benign meningiomas is location-dependent, with intracranial tumors exhibiting a multifactorial recurrence profile, whereas recurrence frequency in spinal tumors is almost exclusively determined by the extent of resection. In addition, we identify sex and tumor status as independent predictors of recurrence only in intracranial WHO grade 1 meningiomas. Of note, observed location-specific effect has not been systematically described previously. Considering the low incidence of spinal meningioma and the eminent impact of the surgical outcome on prognosis, we advocate for treating patients in specialized high-volume centers. To achieve the best possible outcome for patients, including low morbidity and long recurrence-free period, influencing clinical features should be carefully evaluated.

## Conclusion

Spinal meningiomas generally have a more favorable prognosis than intracranial meningiomas, with fewer negative prognostic factors. Our analysis identifies Simpson resection grade ≤ 3 and CNS WHO grade as the strongest predictors of recurrence-free survival. Despite their proximity to highly eloquent neuroanatomical structures, radical resection is often feasible with low morbidity, especially in specialized, experienced high-volume centers. However, it should be emphasized that resection of spinal meningiomas is surgically challenging, and complete resection is not always feasible. For optimal outcomes, complete resection should remain the surgical goal, and treatment should be centralized in experienced institutions.

## Limitations

Limitations include the retrospective design and the lack of follow-up in 18.9% of spinal meningiomas, potentially underestimating the true recurrence rate. Furthermore, the difference in follow-up duration between the groups and the heterogeneity within the group of intracranial meningiomas are also limitations but reflect the real-world setting of our large clinical cohort of consecutive cases. The single-center design and the lack of molecular data advocate for confirmation in an multi-centre trial setting including molecular profiling.

## Supplementary information

Below is the link to the electronic supplementary material.

ESM 1 (DOCX)

## Data Availability

No datasets were generated or analysed during the current study.

## References

[1] Wang JZ et al (2024) Meningioma: International Consortium on Meningiomas consensus review on scientific advances and treatment paradigms for clinicians, researchers, and patients, (in eng). Neuro Oncol 26(10):1742–1780. 10.1093/neuonc/noae082 38695575 10.1093/neuonc/noae082 PMC11449035

[2] Price M et al (2024) CBTRUS Statistical Report: Primary Brain and Other Central Nervous System Tumors Diagnosed in the United States in 2017–2021, (in eng). Neuro Oncol 26(Supplement_6):vi1-vi85. 10.1093/neuonc/noae145 39371035 10.1093/neuonc/noae145 PMC11456825

[3] Riad H, Knafo S, Segnarbieux F, Lonjon N (2013) Spinal meningiomas: surgical outcome and literature review, (in eng). Neurochirurgie 59(1):30–4. 10.1016/j.neuchi.2012.10.137 23395186 10.1016/j.neuchi.2012.10.137

[4] Gottfried ON, Gluf W, Quinones-Hinojosa A, Kan P, Schmidt MH (2003) Spinal meningiomas: surgical management and outcome, (in eng). Neurosurg Focus 14(6):e2.10.3171/foc.2003.14.6.2 15669787 10.3171/foc.2003.14.6.2

[5] Marosi C et al (2008) Meningioma, (in eng). Crit Rev Oncol Hematol 67(2):153–71. 10.1016/j.critrevonc.2008.01.010 18342535 10.1016/j.critrevonc.2008.01.010

[6] Buerki RA, Horbinski CM, Kruser T, Horowitz PM, James CD, Lukas RV (2018) An overview of meningiomas, (in eng). Future Oncol 14(21):2161–2177. 10.2217/fon-2018-0006 30084265 10.2217/fon-2018-0006 PMC6123887

[7] Ravindra VM, Schmidt MH (2023) Spinal meningiomas: Diagnosis, surgical Management, and adjuvant Therapies, (in eng). Neurosurg Clin N Am 34(3):425–435. 10.1016/j.nec.2023.02.007 37210131 10.1016/j.nec.2023.02.007

[8] Goldbrunner R et al (2021) EANO guideline on the diagnosis and management of meningiomas, (in eng). Neuro Oncol 23(11):1821–1834. 10.1093/neuonc/noab150 34181733 10.1093/neuonc/noab150 PMC8563316

[9] Tredway TL, Santiago P, Hrubes MR, Song JK, Christie SD, Fessler RG (2006) Minimally invasive resection of intradural-extramedullary spinal neoplasms, (in eng), *Neurosurgery*, vol. 58, no. 1 Suppl, pp. ONS52-8; discussion ONS52-8, Feb 10.1227/01.neu.0000192661.08192.1c 10.1227/01.neu.0000192661.08192.1c 16479629

[10] Van Goethem JW, van den Hauwe L, Ozsarlak O, De Schepper AM, Parizel PM (2004) Spinal tumors, (in eng). Eur J Radiol 50(2):159–76. 10.1016/j.ejrad.2003.10.021 15081130 10.1016/j.ejrad.2003.10.021

[11] Saraceni C, Harrop JS (2009) Spinal meningioma: chronicles of contemporary neurosurgical diagnosis and management, (in eng). Clin Neurol Neurosurg 111(3):221–6. 10.1016/j.clineuro.2008.10.018 19101080 10.1016/j.clineuro.2008.10.018

[12] Solero CL et al (1989) Spinal meningiomas: review of 174 operated cases, (in eng). Neurosurgery 25(2):153–60 2671779

[13] Maiuri F, De Caro MDB, de Divitiis O, Guadagno E, Mariniello G (Oct2020) Recurrence of spinal meningiomas: analysis of the risk factors, (in eng). Br J Neurosurg 34(5):569–574. 10.1080/02688697.2019.1638886 31290345 10.1080/02688697.2019.1638886

[14] Deska-Gauthier D et al (2024) Clinical, molecular, and genetic features of spinal meningiomas, (in eng). Neurooncol Adv 6(Suppl 3):iii73-iii82. 10.1093/noajnl/vdae123 39430393 10.1093/noajnl/vdae123 PMC11485713

[15] Ricklefs FL et al (Nov 2022) Genetic and epigenetic profiling identifies two distinct classes of spinal meningiomas, (in eng). Acta Neuropathol 144(5):1057–1059. 10.1007/s00401-022-02504-6 36163595 10.1007/s00401-022-02504-6 PMC9547788

[16] Hua L et al (2022) Two predominant molecular subtypes of spinal meningioma: thoracic NF2-mutant tumors strongly associated with female sex, and cervical AKT1-mutant tumors originating ventral to the spinal cord, (in eng). Acta Neuropathol 144(5):1053–1055 10.1007/s00401-022-02474-9 35943573 10.1007/s00401-022-02474-9 PMC9547782

[17] Louis DN et al (2016) The 2016 World Health Organization Classification of Tumors of the Central Nervous System: a summary, (in eng). Acta Neuropathol 131(6):803–20. 10.1007/s00401-016-1545-1 27157931 10.1007/s00401-016-1545-1

[18] Gull HH et al (2022) Spinal Meningioma Surgery through the Ages-Single-Center Experience over Three Decades, (in eng). Medicina (Kaunas) 58(11) 10.3390/medicina58111549 10.3390/medicina58111549 PMC9698896 36363510

[19] Oyemolade TA, Adeolu AA, Malomo AO, Shokunbi MT, Salami AA (2022) Spinal meningioma: clinical profile and outcome of surgical management. Pan Afr Med J 43:44. 10.11604/pamj.2022.43.44.19808 36523272 10.11604/pamj.2022.43.44.19808 PMC9733465

[20] Ucler B, Hergunsel S, Ozturk S, Kozan and M. Kaplan (2018) Simpson grade 2 resection and tumor recurrence in ventrally located spinal meningiomas. Turk Neurosurg 28(6):979–982. 10.5137/1019-5149.Jtn.22416-17.2 29634080 10.5137/1019-5149.JTN.22416-17.2

[21] Krischek B, Goldbrunner R (2023) Paradigm Shift in the Treatment of Meningiomas, (in eng). Adv Exp Med Biol 1416:1–4. 10.1007/978-3-031-29750-2_1 37432615 10.1007/978-3-031-29750-2_1

[22] Goldbrunner R et al (2016) EANO guidelines for the diagnosis and treatment of meningiomas, (in eng). Lancet Oncol 17(9)e383-91. 10.1016/s1470-2045(16)30321-7 10.1016/S1470-2045(16)30321-7 27599143

[23] Kwee LE, Harhangi BS, Ponne GA, Kros JM, Dirven CMF, Dammers R (Nov 2020) Spinal meningiomas: treatment outcome and long-term follow-up, (in eng). Clin Neurol Neurosurg 198:106238. 10.1016/j.clineuro.2020.106238 33096449 10.1016/j.clineuro.2020.106238

[24] Klekamp J, Samii M (1999) Surgical results for spinal meningiomas, (in eng). Surg Neurol 52(6):552–62. 10.1016/s0090-3019(99)00153-6 10660020 10.1016/s0090-3019(99)00153-6

[25] Kobayashi K et al (2021) Clinical features and prognostic factors in spinal meningioma surgery from a multicenter study, (in eng). Sci Rep 11(1):11630. 10.1038/s41598-021-91225-z 34079036 10.1038/s41598-021-91225-z PMC8172892

[26] Jamilson Araújo B, Pereira A, Nogueira de Almeida W, Silva Paiva P (2020) Henrique Pires de Aguiar, M. Jacobsen Teixeira, and S. Kazue Nagahashi Marie, Neuro-oncological features of spinal meningiomas: Systematic review, (in eng). Neurochirurgie 66(1):41-44. 10.1016/j.neuchi.2019.09.027 10.1016/j.neuchi.2019.09.027 31672597

[27] Tominaga H, Kawamura I, Ijiri K, Yone K, Taniguchi N (2021) Surgical results of the resection of spinal meningioma with the inner layer of dura more than 10 years after surgery, (in eng). Sci Rep 11(1):4050. 10.1038/s41598-021-83712-0 33603112 10.1038/s41598-021-83712-0 PMC7893163

[28] Chotai S, Schwartz TH (2022) The Simpson Grading: Is It Still Valid? (in eng). Cancers (Basel) 14(8). 10.3390/cancers14082007 10.3390/cancers14082007 PMC9031418 35454912

[29] Simon M, Gousias K (2024) Grading meningioma resections: the Simpson classification and beyond. Acta Neurochir (Wien) 166(1):28. 10.1007/s00701-024-05910-9 38261164 10.1007/s00701-024-05910-9 PMC10806026

[30] Spille DC et al (2021) Risk of tumor recurrence in intracranial meningiomas: comparative analyses of the predictive value of the postoperative tumor volume and the Simpson classification. J Neurosurg 134(6):1764–1771. 10.3171/2020.4.Jns20412 32679565 10.3171/2020.4.JNS20412

[31] Brokinkel B et al (2021) The Simpson grading: defining the optimal threshold for gross total resection in meningioma surgery. Neurosurg Rev 44(3):1713–1720. 10.1007/s10143-020-01369-1 32809081 10.1007/s10143-020-01369-1 PMC8397672

[32] Black PM (1993) Meningiomas. Neurosurgery 32(4):643–57. 10.1227/00006123-199304000-00023 8474655 10.1227/00006123-199304000-00023

[33] Bumrungrachpukdee P, Pruphetkaew N, Phukaoloun M, Pheunpathom N (2014) Recurrence of intracranial meningioma after surgery: analysis of influencing factors and outcome, (in eng). J Med Assoc Thai 97(4):399–406 24964682

[34] Mirimanoff RO, Dosoretz DE, Linggood RM, Ojemann RG, Martuza RL (1985) Meningioma: analysis of recurrence and progression following neurosurgical resection. J Neurosurg 62(1):18–24. 10.3171/jns.1985.62.1.0018 3964853 10.3171/jns.1985.62.1.0018

[35] Garufi G, Scalia G, Ricciardo G, Umana GE, Conti A, Cardali SM (2024) Spinal intramedullary meningiomas: a systematic review with a case illustration. World Neurosurg 187:11–18. 10.1016/j.wneu.2024.03.124 38548054 10.1016/j.wneu.2024.03.124

[36] Perven G, Entezami P, Gaudin D (2015) A rare case of intramedullary “whorling-sclerosing” variant meningioma. Springerplus 4:318. 10.1186/s40064-015-1110-8 26155457 10.1186/s40064-015-1110-8 PMC4491092

[37] Dang DD, Mugge LA, Awan OK, Gong AD, Fanous AA (2024) Spinal meningiomas: a comprehensive review and update on advancements in molecular characterization, diagnostics, surgical approach and technology, and alternative therapies. Cancers (Basel). 10.3390/cancers16071426 38611105 10.3390/cancers16071426 PMC11011121

[38] Hua L et al (2018) Clinical and prognostic features of spinal meningioma: a thorough analysis from a single neurosurgical center. J Neurooncol 140(3):639–647. 10.1007/s11060-018-2993-3 30209689 10.1007/s11060-018-2993-3

[39] Driver J, Santagata S, Bi WL, Chi JH (2023) Genomic analysis of spinal meningiomas: correlation with histopathological grade. J Neurosurg Spine 39(6):729–733. 10.3171/2023.6.Spine23425 37728381 10.3171/2023.6.SPINE23425

