# Supplementary material for: Predictive factors in spinal meningiomas – a comparative analysis with intracranial meningiomas of a high-volume skull and base center
Source: Neurosurg Rev. 2026 Feb 7;49(1):206. doi: 10.1007/s10143-026-04151-x (PMC12882958; doi:10.1007/s10143-026-04151-x)
Supplement: Supplementary file 1 — (DOCX) [file 10143_2026_4151_MOESM1_ESM.docx]

**Supplementary tables:**

Supplementary table 3: Logistic regression analyses of rf risk factors for the development of recurrence. The asterisk indicates statistical significance. Likelihood ratio tests were used for overall effects; Wald-based confidence intervals are reported where applicable. For spinal meningiomas, some confidence intervals could not be reliably estimated due to limited event numbers.

|  | Parameter | Odds ratio (95% CI) | p-value |
| --- | --- | --- | --- |
| Intracranial meningiomas | WHO classification | 2 vs. 1 4.85 (3.66-6.42)  3 vs. 1 48.15 (10.94-212.0)  3 vs. 2 9.94 (2.24-44.14) | <0.0001*  <0.0001*  0.0025* |
|  | Simpson classification | ≤3 vs. >3 0.24 (0.19-0.31)  >3 vs. ≤3 4.17 (3.23-5.39) | <0.0001*  <0.0001* |
| Spinal meningiomas | WHO classification | 2 vs. 1 -  3 vs. 1 -  3 vs. 2 - | 0.369  0.027*  0.033* |
|  | Simpson classification | ≤3 vs. >3 0.06 (0.01-0.22) | <0.0001* |

Supplementary table 4: Multivariate logistic regression analyses restricted to WHO grade 1 meningiomas. Likelihood ratio tests were used to assess the independent contribution of each variable to tumor recurrence.

| Parameter | Intracranial meningiomas | | Spinal meningiomas | |
| --- | --- | --- | --- | --- |
|  | LR χ² | p-value | LR χ² | p-value |
| Extent of resection  (Simpson ≤ 3) | 64.04 | <0.0001* | 11.72 | 0.0006* |
| Sex | 10.53 | 0.0012* | 2.70 | 0.100 |
| Tumor status  (primary vs. recurrent) | 25.54 | <0.0001* | 2.32 | 0.128 |

Table 5: Multivariate logistic regression analyses were performed separately for intracranial and spinal meningiomas. Likelihood ratio tests were used to assess the independent contribution of each variable to recurrence. The effect of adjuvant radiotherapy in spinal meningiomas could not be reliably estimated due to limited event numbers.

| Parameter | Intracranial meningiomas | | Spinal meningiomas | |
| --- | --- | --- | --- | --- |
|  | LR χ² | p-value | LR χ² | p-value |
| Extent of resection  (Simpson ≤ 3) | 76.28 | <0.0001* | 11.72 | 0.0006* |
| Sex | 8.13 | 0.0044* | 2.70 | 0.100 |
| Tumor status  (primary vs. recurrent) | 51.61 | <0.0001* | 2.32 | 0.128 |
| WHO classification | 71.89 | <0.0001* | 1.91 | 0.385 |
| Adjuvant Radiotherapy | 36.16 | <0.0001* | - | - |
